# Analysis of vestibular-balance symptoms according to symptom duration: dimensionality of the Vertigo Symptom Scale-short form

**DOI:** 10.1186/s12955-015-0207-7

**Published:** 2015-01-22

**Authors:** Masaki Kondo, Kensuke Kiyomizu, Fumiyuki Goto, Tadashi Kitahara, Takao Imai, Makoto Hashimoto, Hiroaki Shimogori, Tetsuo Ikezono, Meiho Nakayama, Norio Watanabe, Tatsuo Akechi

**Affiliations:** Department of Psychiatry and Cognitive-Behavioral Medicine, Nagoya City University Graduate School of Medical Sciences, Nagoya, Aichi Japan; Department of Psychiatry, Yoshida Hospital, Nobeoka, Miyazaki Japan; Department of Otolaryngology, Head and Neck Surgery, Faculty of Medicine, University of Miyazaki, Miyazaki, Japan; Department of Otorhinolaryngology, National Hospital Organization, Tokyo Medical Center, Meguro, Tokyo Japan; Department of Otolaryngology-Head and Neck Surgery, Osaka University Graduate School of Medicine, Suita, Osaka Japan; Department of Otorhinolaryngology-Head and Neck Surgery, Nara Medical University, Kashihara, Nara Japan; Department of Otolaryngology, Yamaguchi University Graduate School of Medicine, Ube, Yamaguchi Japan; Department of Otolaryngology, Saitama Medical University, Iruma, Saitama Japan; Department of Otolaryngology, Nagoya City University Graduate School of Medical Sciences, Nagoya, Aichi Japan; Department of Clinical Epidemiology, Translational Medical Center, National Center of Neurology and Psychiatry, Kodaira, Tokyo Japan

**Keywords:** Vestibular disease, Psychosomatic medicine, Vertigo, Dizziness, Anxiety, Factor analysis, Validity, Reliability, Japanese

## Abstract

**Background:**

Dizziness or vertigo is associated with both vestibular-balance and psychological factors. A common assessment tool is the Vertigo Symptom Scale (VSS) -short form, which has two subscales: vestibular-balance and autonomic-anxiety. Despite frequent use, the factor structure of the VSS-short form has yet to be confirmed. Here, we clarified the factor structure of the VSS-short form, and assessed the validity and reliability of the Japanese version of this tool.

**Methods:**

We conducted a cross-sectional, multicenter, psychometric evaluation of patients with non-central dizziness or vertigo persisting for longer than 1 month. Participants completed the VSS-short form, the Dizziness Handicap Inventory, and the Hospital Anxiety and Depression Scale. They also completed the VSS-short form a second time 1–3 days later. The questionnaire was translated into Japanese and cross-culturally adapted. We conducted a confirmatory factor analysis followed by an exploratory factor analysis. Convergent and discriminant validity, internal consistency, and test-retest reliability were evaluated.

**Results:**

The total sample and retest sample consisted of 159 and 79 participants, respectively. Model-fitting for a two-subscale structure in a confirmatory factor analysis was poor. An exploratory factor analysis produced a three-factor structure: long-duration vestibular-balance symptoms, short-duration vestibular-balance symptoms, and autonomic-anxiety symptoms. Regarding convergent and discriminant validity, all hypotheses were clearly supported. We obtained high Cronbach’s α coefficients for the total score and subscales, ranging from 0.758 to 0.866. Total score and subscale interclass correlation coefficients for test-retest reliability were acceptable, ranging from 0.867 to 0.897.

**Conclusions:**

The VSS-short form has a three-factor structure that was cross-culturally well-matched with previous data from the VSS-long version. Thus, it was suggested that vestibular-balance symptoms can be analyzed separately according to symptom duration, which may reflect pathophysiological factors. The VSS-short form can be used to evaluate vestibular-balance symptoms and autonomic-anxiety symptoms, as well as the duration of vestibular-balance symptoms. Further research using the VSS-short form should be required in other languages and populations.

## Background

Vestibular-balance symptoms, such as vertigo and dizziness, affect approximately 20% of the population [1,2]. These often become chronic and can greatly impair daily living [2,3]. Vestibular-balance symptoms are often accompanied and interact with psycho-physiological symptoms, especially anxiety, which may also have a great impact on patient’s quality of life [4-6]. Therefore, the clinical state of patients with vestibular-balance symptoms cannot be completely evaluated with vestibular and balance function tests, such as caloric tests or posturography [7,8]. Patient-reported scales assessing both vestibular-balance and psycho-physiological factors are absolutely necessary to evaluate severity of symptoms or effectiveness of treatment, such as vestibular rehabilitation [9] and cognitive behavioral therapy [10].

Two patient-reported scales have been widely used to comprehensively evaluate patients with vestibular-balance symptoms: the Dizziness Handicap Inventory (DHI) [7] and the Vertigo Symptom Scale (VSS) [8]. The DHI evaluates handicaps due to dizziness in daily life. The VSS, which assesses patient-reported symptoms, has an advantage over the DHI in that it is not just used to evaluate the frequency of vestibular-balance symptoms, but also the severity of autonomic-anxiety symptoms, which have a great impact on quality of life. The VSS has the highest comprehensive validity of the self-rated scales assessing vertigo- or dizziness-related symptoms [11]. There are two versions of the VSS: the long version (VSS-lv) and the short form (VSS-sf). The VSS-lv includes 34 items that were created on the basis of patient interviews. It can be used to measure the symptom frequency over the past year, which is useful for epidemiological research. Principal component analysis of the VSS-lv reveals a three-factor structure: acute attack of vertigo or vestibular-balance symptom of long duration, vertigo of short duration or vestibular-balance symptom of short duration, and autonomic-anxiety symptom. From an empirical perspective, the first and second factors can be combined into one subscale, such that the VSS-lv is defined as consisting of two subscales: the Vestibular-balance subscale and the Autonomic-anxiety subscale [8]. For consistency between research groups, the two-factor solution has been employed continuously, although the optimal number of factors was not examined or determined to be ‘three factors’ [12-14].

The VSS-sf has 15 items derived from the VSS-lv. However, the factor structure has not been thoroughly confirmed. The VSS-sf was developed to measure symptom frequency over 1 month, with the main goal of assessing therapeutic effect, and has been used in clinical trials [9,15]. Although the VSS-sf has been defined as having two subscales, similar to the VSS-lv [16], a previous study failed to verify a simple two-factor structure for the VSS-sf via factor analysis. In the study, the Kaiser criterion and scree plot were employed to determine the number of factors, and these yielded a two- and three-factor solution, respectively. Ultimately, a two-factor solution was adopted [17] for consistency with previous studies. However, recent guidelines recommend the use of multiple methods that are better able to determine the number of factors than the Kaiser criterion [18,19]. Thus, our primary aim was to clarify the factor structure of the VSS-sf using the recent recommendations for factor analysis of Classical Test Theory (CTT) [18,19]. We used factor analysis of CTT not methods of Item Response Theory (IRT) because both CTT and IRT are acceptable in COSMIN checklist, which is the recent guideline for designing or reporting a study on measurement properties [20]. Our secondary aim was to confirm the validity and reliability of the VSS-sf Japanese version used in the present study.

## Methods

### Procedure and participants

We conducted a cross-sectional, multicenter, psychometric evaluation. Participants were recruited from the otolaryngology departments of four university hospitals and two general hospitals (three in urban areas, two in suburbs, and one in a rural area) in Japan from February to September of 2013. Eligible participants included both outpatients and inpatients with vertigo or dizziness persisting for longer than 1 month. All participants were at least 20 years of age and were native Japanese speakers. Neuro-otology experts excluded patients with vertigo or dizziness caused by the central nervous system, and diagnosed all patients according to the Diagnostic Guideline of Equilibrium Disorders by Japan Society for Equilibrium Research. The Japan Society for Equilibrium Research has strong relation with the Barany Society (the International Society for Neuro-Otology), and the definitions of vestibular diseases are nearly similar between these two societies [21].

All participants completed three self-administered questionnaires, which included socio-demographic variables, on site. Participants who consented to the retest study received an additional VSS-sf questionnaire, and were instructed to complete it 1–3 days after the initial session. They were asked to record the precise date of completion. Outpatients and inpatients returned the retest questionnaire by mail and by hand, respectively. With respect to test-retest reliability in the current study population, we considered 1–3 days to be an appropriate interval for completion because 1) vertigo- or dizziness-related symptoms can vary widely on the scale of several days, and 2) because 24- or 48-hour intervals have been adopted in previous studies for similar populations [17,22].

### Measurements

#### Vertigo Symptom Scale–short form (VSS-sf)

The Vertigo Symptom Scale-short form (VSS-sf) is a 15-item, self-report instrument that measures the frequency of vertigo, dizziness, unsteadiness, and concomitant autonomic/anxiety symptoms over the past month (Table 1). The VSS-sf uses a five-point Likert scale: 0 (never), 1 (a few times), 2 (several times), 3 (quite often [every week]), and 4 (very often [most days]). The total score ranges from 0 to 60, and a higher score indicates a higher frequency of symptoms. The VSS-sf has two subscales: the Vestibular-balance subscale (VSS-sf-V) and the Autonomic-anxiety subscale (VSS-sf-A), although the VSS-sf comprises items extracted from the VSS-lv, which has a three-factor structure [8]. Both the internal consistency and test-retest reliability of the original English version of the VSS have been established [23,24]. The reliability and validity of the Norwegian and Turkish versions have also been verified [17,22].

**Table 1:** **Distribution profile of the vertigo symptom scale-short form in the total sample (n = 159)**

	**Item**	**Mean ± SD**	**Skewness**	**Kurtosis**	**Lowest (%)**	**Highest (%)**
**Vertigo-balance subscale score (VSS-sf-V)**		10.1 ± 7.85	0.71	−0.32	8.2	0.6
1	A feeling that either you, or things around you, are spinning or moving, lasting less than 20 minutes	1.53 ± 1.45	0.45	−1.21	34.0	13.8
3	Nausea (feeling sick), vomiting	0.96 ± 1.25	1.14	0.05	52.2	5.7
4	A feeling that either you, or things around you, are spinning or moving, lasting more than 20 minutes	0.99 ± 1.36	1.20	0.07	54.1	10.1
6	A feeling of being dizzy, disorientated or swimmy, lasting all day	1.53 ± 1.58	0.52	−1.32	39.0	20.8
8	Unable to stand or walk properly without support, veering or staggering to one side	1.13 ± 1.39	0.96	−0.45	48.4	10.7
10	Feeling unsteady, about to lose balance, lasting more than 20 minutes	0.89 ± 1.29	1.19	0.06	60.4	6.3
13	Feeling unsteady, about to lose balance, lasting less than 20 minutes	1.32 ± 1.37	0.69	−0.78	39.0	10.7
15	A feeling of being dizzy, disorientated or swimmy, lasting less than 20 minutes	1.72 ± 1.47	0.33	−1.30	27.0	18.9
**Autonomic-anxiety subscale score (VSS-sf-A)**		5.34 ± 4.96	1.11	0.95	17.0	0.0
2	Hot or cold spells	1.20 ± 1.40	0.81	−0.70	47.2	10.7
5	Heart pounding or fluttering	0.82 ± 1.17	1.35	0.76	57.2	4.4
7	Headache, or feeling of pressure in the head	1.42 ± 1.34	0.62	−0.79	32.1	11.3
9	Difficulty breathing, short of breath	0.64 ± 1.10	1.80	2.34	66.0	4.4
11	Excessive sweating	0.58 ± 1.08	1.94	2.82	69.2	3.8
12	Feeling faint, about to black out	0.46 ± 0.90	**2.09**	3.76	73.6	1.3
14	Pains in the heart or chest region	0.21 ± 0.59	**3.31**	**11.66**	84.9	0.0
**Total score (VSS-sf-T)**		15.4 ± 11.3	0.86	0.30	3.1	0.0

SD: Standard Deviation; Skewness > 2.0 or kurtosis > 7.0 are in bold.

#### Dizziness Handicap Inventory (DHI)

The Dizziness Handicap Inventory (DHI) is a 25-item, self-report questionnaire that evaluates the impact of dizziness or vertigo on quality of life, and can be used to assess the severity and effect of therapeutic treatments. Each item is rated on a three-point scale. A higher total score reflects a more severe handicap. The DHI comprises items on three subscales: Emotional (DHI-E: anxiety or mental stress influenced by dizziness or vertigo), Functional (DHI-F: disability affecting daily living caused by dizziness or vertigo), and Physical (DHI-P: dizziness or vertigo provoked by specific self-motions). We employed the original three-subscale structure from various factorial solutions of the DHI [25]. The reliability and validity of the original English and Japanese versions have been established [7,26].

#### Hospital Anxiety and Depression Scale (HADS)

The Hospital Anxiety and Depression Scale (HADS) is a 14-item scale that evaluates general anxiety and depression in patients in a non-psychiatric hospital setting. It comprises the Anxiety subscale (HADS-A) and the Depression subscale (HADS-D), which both have seven items on a four-point scale. Higher subscale scores indicate higher levels of either anxiety or depression. The reliability and validity of the HADS have been documented for both the original and the Japanese version [27,28]. A score of eight or above on the HADS-A and HADS-D reflects the presence of anxiety disorders and depression, respectively [29].

### Translation and cross-cultural adaptation

The translation procedure involved four steps. First, two native Japanese speakers independently translated the original English version into Japanese. One of the translators was a psychiatrist who was familiar with persistent dizziness, while the other was a neuro-otologist who specializes in somatic medicine. Second, the two translators met with a Japanese methodologist who specializes in psychiatric clinical research to discuss the forward-translated Japanese version. Third, the test was translated back into English by a neuro-otology specialist who was blind to the original version. The back-translator spoke both Japanese and English, and was advised by a native English speaker who was also blind to the original version. A set of guidelines regarding the translation of health measurement instruments has recommended that one translator have expertise on the construct to be measured while the other has language expertise but naivety regarding the topic [20]. However, we chose the translators among a group of medical doctors who were familiar with vestibular-balance symptoms, because the Japanese word “memai” encompasses a wide range of concepts related to vestibular-balance symptoms (e.g. vertigo, dizziness, unsteadiness, faintness, and lightheadedness) and is thus difficult for non-medical translators to translate forward or back correctly. Finally, the author of the original English version confirmed semantic and conceptual equivalence between the original and the back-translated English version. In a meeting with five psychiatrists, a psychiatric nurse, and a psychiatric methodologist, all translation steps were approved and the provisional Japanese version was declared to be idiomatic and conceptually equivalent to the original English version of the test.

Next, for cross-cultural adaptation, the provisional Japanese version was tested on six Japanese patients with persistent dizziness pre- and post-cognitive behavioral therapy. Three of the patients reported that the phrasing of two anchor points (the Japanese translation of “quite often” and “very often”) were difficult to distinguish from one another. In fact, these phrases did not reflect therapeutic changes in the three patients, even though the frequency of their symptoms was clearly improved. An additional meeting of the group of experts described above concluded that the meanings of these original English phrases were difficult to describe appropriately using simple Japanese words. Consequently, we added concrete numeric values for each anchor point, which we obtained from the author of the original version: 0 (None), 1 (A few times [approximately 1–3 times]), 2 (Several times [approximately 4–6 times]), 3 (frequent [more than approximately seven times in less than approximately 14 days]), and 4 (extremely frequent [during a period of approximately 15 days]). Another group of six Japanese patients with persistent dizziness and similar treatment conditions were able to understand these fixed expression anchor points with no difficulty. Total scores on the VSS-sf seemed to clearly reflect patient therapeutic changes. Thus, we conclusively defined the final Japanese version of the VSS-sf.

### Data screening and floor/ceiling effects

Cases with outlying or missing values were excluded. We checked whether each item in the VSS-sf was normally distributed. Guidelines suggested that we could assume a normal distribution when skewness and kurtosis were no greater than 2.0 and 7.0, respectively [18]. Floor or ceiling effects are considered to be present when more than 15% of participants achieve the lowest or highest possible scores [30].

### Dimensionality (structural validity)

First, we conducted a confirmatory factor analysis (CFA) assuming a two-factor model, which corresponds to two subscales: VSS-sf-V and VSS-sf-A. We used a maximum likelihood method assuming that each VSS-sf item followed a normal distribution. Otherwise, we used a generalized least square method. We examined the following model fit criteria: standardized root mean square residual (SRMR), comparative fit index (CFI), and root mean square error of approximation (RMSEA). Guidelines suggested that an SRMR equal to 0.08 or below, CFI equal to 0.95 or above, and RMSEA equal to 0.05 or below are indicative of good fit [18].

If the results of the CFA suggested an insufficient model fit, we conducted an exploratory factor analysis (EFA). To evaluate the suitability of the data for EFA, we calculated Bartlett’s test for sphericity and the Kaiser–Meyer–Olkin measure of sampling adequacy (KMO), which should exceed the recommended minimum value of 0.60. To determine the optimal number of factors, multiple criteria were examined. These included the Kaiser criterion (“eigenvalue greater than one” rule), scree plot, parallel analysis, minimum average partial (MAP) procedure, and Bayesian information criterion (BIC). We calculated both the MAP and BIC values for a one-factor to seven-factor solution, and calculated the number of factors in which each value was minimized. Guidelines suggested that researchers should use multiple methods to determine the number of factors and should emphasize methods known to perform better (such as parallel analysis and the MAP procedure) more than those known to provide biased estimates (such as the Kaiser criterion) [18,19]. Because we assumed that there would be interfactor correlations, we used oblique rotations: the direct Oblimin rotation and the Promax rotation.

### Other psychometric properties

For construct validity (convergent/discriminant validity), we examined the following three hypotheses with Pearson correlation coefficients to evaluate univariate relationships. Regarding the strength of relationships, we followed several suggestions that the relationship should be regarded respectively as large, moderate, and weak if the correlation coefficiant was around ±0.50, ±0.30, and ±0.10 [31,32].

- Hypothesis 1: The total score on the VSS-sf (VSS-sf-T) should have a large positive correlation (r > 0.50) with the DHI total score (DHI-T) because the frequency of vertigo-balance symptoms and coexisting autonomic-anxiety symptoms is expected to relate to handicaps resulting from dizziness.
- Hypothesis 2: The VSS-sf-V score should have a large positive correlation (r > 0.50) with the DHI-T, and a moderate or weak positive correlation (0.10 < r < 0.50) with the HADS-D. We assumed that the frequency of vertigo and dizziness was related to handicaps resulting from dizziness, and to a smaller degree, depressive symptoms.
- Hypothesis 3: The VSS-sf-A score should have a large positive correlation (r > 0.50) with the HADS-A, and a moderate or weak positive correlation (0.10 < r < 0.50) with the DHI-P. We supposed that the frequency of autonomic-anxiety symptoms associated with vertigo and dizziness was related to general levels of anxiety, and to a smaller degree, the severity of dizziness provoked by self-motion.

Internal consistency is considered to be good when Cronbach’s α coefficient is between 0.70 and 0.95 [30]. We calculated Cronbach’s α coefficients for the VSS-sf-T, VSS-sf-V, and VSS-sf-A.

Regarding the test-retest reliability (reproducibility) of the VSS-sf-T, VSS-sf-V, and VSS-sf-A, we calculated interclass correlation coefficients (ICC) as a measure of absolute agreement for single measures under a two-way random model. An ICC of 0.70 is recommended as a minimum standard for reliability [30]. As a sensitivity analysis to examine the effect of the retest interval, we also calculated ICCs for two subgroups: a 1-day interval subgroup and a 2- or 3-day interval subgroup.

### Sample size, statistical analysis, and ethics

We set the sample size at 150 because the adequate subject-to-variable ratio for factor analysis has been known empirically to range from 4:1 to 10:1, with a minimum of 100 subjects [20]. The target size of the retest sample was greater than 50 [30].

We used three methods to determine the number of factors in the EFA (parallel analysis, MAP procedure, and BIC), which were conducted with R ver. 3.0.2 (The R Foundation for Statistical Computing, Vienna, Austria) and EZR ver. 1.24 (Saitama Medical Center, Jichi Medical University, Saitama, Japan) [33], which is a graphical user interface for R. CFA was performed with SPSS Amos for Windows ver. 22.0 J. All other analyses were performed with SPSS Statistics for Windows ver. 22.0 J. A two-tailed P of less than 0.05 was considered to be significant.

The ethical committee of Nagoya City University Graduate School of Medical Sciences and similar ethical review boards at all other study sites approved the study, which followed the guidelines set by the Helsinki Declaration. Written informed consent was obtained from all participants.

## Results

### Data quality and floor/ceiling effects

A total of 191 patients completed the questionnaires. Excluding cases with missing values, the total sample of the study contained 159 participants (83.2%). We found no influential outliers and no systematic differences between the total sample and the excluded cases. The retest sample contained 79 participants. Table 2 summarizes the demographic characteristics and clinical status of the total and retest samples. Table 1 shows how two items of the VSS-sf were considered not to follow a normal distribution and that neither the floor nor ceiling effect were observed in the total and subscale scores, except for the floor effect in the VSS-sf-A.

**Table 2:** **Demographic characteristics and clinical status of the study sample**

	**Total sample (n = 159)**		**Retest sample (n = 79)**	
	**n**	**(%)**	**n**	**(%)**
**Male**	55	(34.6)	28	(35.4)
Age (year)*	57.4	±16.8	57.5	±16.4
**Marital status**				
Married	106	(66.7)	51	(64.6)
Unmarried/Divorced/Widowed	53	(33.3)	28	(35.4)
**Educational status**				
Junior high school/High school	93	(58.5)	46	(58.2)
Junior college/University	66	(41.5)	33	(41.8)
**Occupational status**				
Full-time worker	51	(32.1)	28	(35.4)
Part-time worker	14	(8.8)	4	(5.1)
Housewife	42	(26.4)	20	(25.3)
Student	1	(0.6)	1	(1.3)
Others	51	(32.1)	26	(32.9)
**Diagnosis**				
Ménière’s disease	41	(25.8)	20	(25.3)
Benign paroxysmal positional vertigo	16	(10.1)	9	(11.4)
Vertebrobasilar artery insufficiency	13	(8.2)	5	(6.3)
Vestibular neuronitis	12	(7.5)	9	(11.4)
Sudden deafness with vertigo or dizziness	5	(3.1)	3	(3.8)
Vestibular migraine	3	(1.9)	2	(2.5)
Acoustic nerve tumor	3	(1.9)	1	(1.3)
Other vestibular diseases^†^	14	(8.8)	10	(12.7)
Vertigo or dizziness without any other vestibular disease^‡^	52	(32.7)	20	(25.3)
Disease duration (month)*	57.9	±72.5	61.3	±68.2
**Inpatient**	31	(19.5)	26	(32.9)
**Mental status**				
Clinical anxiety (HADS-A ≥ 8)	56	(35.2)	30	(38.0)
Clinical depression (HADS-D ≥ 8)	64	(40.3)	32	(40.5)

*Mean ± SD. ^†^Vestibular diseases which are not defined by the diagnostic guideline of Japan Society for Equilibrium Research, such as unexplained vestibular dysfunction, age-related vestibular dysfunction. ^‡^Including psychogenic dizziness without any other vestibular disease. HADS-A/D: Hospital Anxiety and Depression Scale-Anxiety/Depression subscale.

### Dimensionality (structural validity)

We used the generalized least square method for both the CFA and EFA because the VSS-sf had two non-normally distributed variables. All model-fit indices for the CFA examining the assumed two-factor simple structure showed a poor model fit: the SRMR, CFI, and RMSEA were 0.1175, 0.407, and 0.093 (90% confidence interval: 0.077–0.110), respectively.

Thus, we conducted an EFA. Bartlett’s test for sphericity was significant (p < 0.001) and the KMO was 0.80, which confirmed that the data were suitable for EFA. Regarding the number of factors, the results of the Kaiser criterion, scree test, parallel analysis, and BIC indicated that a three-factor solution was the best fit. Although the MAP value in a two-factor solution was the smallest, the MAP values in solutions from one- to three-factors were nearly equal to each other. Therefore, we comprehensively concluded that the optimal number of factors was three. We assessed the factor pattern and factor structure matrix with commonality and interfactor correlations from EFA using a direct Oblimin rotation with delta = 0 (Table 3). We confirmed that a Promax rotation provided similar solutions. One of three factors corresponded exactly to the VSS-sf-A subscale. The other two factors appeared to comprise four “vestibular-balance short-duration symptom” items and four “vestibular-balance long-duration symptom” items in the VSS-sf-V, with two instances of cross-loading. The correlation matrix subjected to EFA is available upon request.

**Table 3:** **Factor pattern/structure matrix, commonality, and interfactor correlation in exploratory factor analysis**

		**Pattern coefficient**			**Structure coefficient**			
	**Outline of item**	**F1**	**F2**	**F3**	**F1**	**F2**	**F3**	***h*** ^**2**^
**Vertigo-balance subscale**								
10	Unsteadiness (>20 minutes)	**0.868**	0.000	0.044	**0.887**	−0.416	0.413	0.838
4	Vertigo (>20 minutes)	**0.742**	−0.103	−0.069	**0.760**	−0.422	0.281	0.742
3	Nausea, vomiting	**0.463**	0.048	0.182	**0.518**	−0.227	0.362	0.469
6	Dizziness (all day)	**0.427**	**−0.364**	0.093	**0.634**	**−0.592**	0.397	0.673
15	Dizziness (<20 minutes)	−0.160	**−0.947**	0.093	0.317	**−0.905**	0.344	0.865
1	Vertigo (<20 minutes)	0.081	**−0.746**	−0.092	0.386	**−0.752**	0.193	0.781
13	Unsteadiness (<20 minutes)	0.077	**−0.672**	0.111	0.435	**−0.745**	0.370	0.665
8	Difficulty in standing or walking	**0.372**	**−0.427**	0.016	**0.576**	**−0.604**	0.318	0.612
**Autonomic-anxiety subscale**								
9	Shortness of breath	−0.177	−0.062	**0.838**	0.207	−0.262	**0.784**	0.693
14	Chest pain	−0.035	0.140	**0.693**	0.195	−0.078	**0.631**	0.520
5	Heart pounding	0.169	0.000	**0.504**	0.382	−0.247	**0.575**	0.521
12	Feeling faint	0.193	−0.035	**0.500**	0.421	−0.292	**0.594**	0.477
2	Chills or hot flushes	0.147	−0.041	**0.472**	0.366	−0.267	**0.548**	0.487
11	Excessive sweating	−0.044	−0.127	**0.461**	0.210	−0.262	**0.485**	0.511
7	Headache	0.167	−0.099	**0.351**	0.361	−0.294	**0.455**	0.391
	**Interfactor correlation**	F1	F2	F3				
	F1							
	F2	−0.461						
	F3	0.424	−0.337					

Exploratory factor analysis used the generalized least square method, direct Oblimin rotation with delta = 0. Factor pattern/structure coefficients of 0.300/0.450 or more, respectively, are in bold. Factor 1 (F1): Vestibular-balance long-duration symptom; Factor 2 (F2): Vestibular-balance short-duration symptom; Factor 3 (F3): Autonomic-anxiety symptom; *h* ^2^: commonality.

### Other psychometric properties

The total and subscale scores of the three questionnaires were considered to be normally distributed because of their skewness < 2.0, kurtosis < 7.0, and distribution graphs. Therefore, we employed the Pearson correlation coefficient to assess the construct validity. The correlation matrix for the convergent/discriminant validity is shown in Table 4, supporting all three hypotheses clearly. Regarding internal consistency, the Cronbach’s α coefficient for the VSS-sf-T, VSS-sf-V, and VSS-sf-A were considered to be good: 0.866, 0.853, and 0.758, respectively. The ICC values for test-retest reliability are shown in Table 5. The ICC values in the retest sample and each subgroup were acceptable.

**Table 4:** **Construct validity: Pearson correlation coefficients between variables**

	**VSS-sf-T**	**VSS-sf-V**	**VSS-sf-A**	**DHI-T**	**DHI-E**	**DHI-F**	**DHI-P**	**HADS-A**	**HADS-D**
VSS-sf-T									
VSS-sf-V	0.926								
VSS-sf-A	0.803	0.519							
DHI-T	**0.557**	**0.521**	0.440						
DHI-E	0.487	0.434	0.418	0.906					
DHI-F	0.508	0.474	0.402	0.935	0.806				
DHI-P	0.485	0.482	**0.339**	0.799	0.554	0.634			
HADS-A	0.558	0.425	**0.595**	0.555	0.623	0.438	0.401		
HADS-D	0.484	**0.390**	0.480	0.534	0.590	0.467	0.337	0.746	

All correlations are significant (*p* < 0.001). Values in bold indicate the relationships mentioned in the prior hypotheses. VSS-sf-T/V/A: Vertigo Symptom Scale-short form Total score/Vestibular-balance subscale/Autonomic-anxiety subscale; DHI-T/E/F/P: Dizziness Handicap Inventory-Total score/Emotional subscale/Functional subscale/Physical subscale; HADS-A/D: Hospital Anxiety and Depression Scale-Anxiety subscale/Depression subscale.

**Table 5:** **Intraclass correlation coefficients for test-retest reliability**

	**1 to 3-day interval (n = 79)**	**1-day interval (n = 45)**	**2 or 3-day interval (n = 34)**
VSS-sf-T	0.888 [0.829-0.927]	0.921 [0.861-0.956]	0.848 [0.717-0.921]
VSS-sf-V	0.867 [0.800-0.913]	0.899 [0.823-0.943]	0.833 [0.693-0.913]
VSS-sf-A	0.897 [0.839-0.934]	0.920 [0.853-0.956]	0.869 [0.756-0.932]

[ ]: 95% confidence interval. VSS-sf-T/V/A: Vertigo Symptom Scale-short form Total score/Vestibular-balance subscale/Autonomic-anxiety subscale.

## Discussion

The present study is, to the best of our knowledge, the first to clarify the dimensionality of the VSS-sf in a manner recommended by the recent guidelines for factor analysis [18-20]. Our most important finding was that the VSS-sf in our Japanese sample consisted of three factors that were well-matched with the three factors in the VSS-lv in British, Mexican, and German samples [8,12,13]. These results suggest that the symptoms of patients with vertigo or dizziness may consist of three factors with cross-cultural consistency: the Vestibular-balance long-duration symptom, Vestibular-balance short-duration symptom, and Autonomic-anxiety symptom.

The first well-known self-administered questionnaire for measuring vestibular-balance symptoms is the DHI, which Jacobson developed in 1990 to evaluate the extent of handicaps due to dizziness [7]. Around the same time, in 1992, Yardley developed the VSS, which has an advantage in that it can be used to evaluate the frequency of not only vestibular-balance symptoms but also autonomic-anxiety symptoms, which have a strong impact on quality of life. At the time, Yardley made the important point that the VSS-lv has a three-factor structure: the Vestibular-balance long-duration symptom, Vestibular-balance short-duration symptom, and Autonomic-anxiety symptom [8]. Nevertheless, the two-subscale structure has been defined from the perspective of empirical observation, and has been employed widely since then. In two following principle component analysis studies, the VSS-lv was defined as consisting of two factors even though a three-factor solution was indicated by a scree plot, which was the only reliable method for determining the appropriate number of factors in these studies [12,13]. Moreover, a following study analyzed the VSS-lv using a two-factor solution without examining the number of factors [14]. It appears that the empirically determined two-subscale structure has had a huge effect on the following methodologies in studies regarding the dimensionality of the VSS-lv and the VSS-sf. We suggest that both the VSS-lv and VSS-sf be analyzed not as empirical two-factor solutions but as statistics-based three-factor solutions, at least in clinical research.

We further suggest that vestibular-balance symptoms be analyzed separately, according not only to the symptom aspects (vertigo, dizziness, and unsteadiness) but also the symptom durations (vestibular-balance symptom lasting more [or less] than 20 minutes), which may better reflect pathophysiological factors behind the symptoms. For example, there are items in the VSS-lv that assess vertigo, dizziness, and unsteadiness lasting less than 2 minutes, and these items did not load on the Vestibular-balance factor but did load on the Autonomic-anxiety factor [12]. In the present study, the factor on which the items of vertigo, dizziness, and unsteadiness loaded depended on whether the symptom duration was more or less than 20 minutes. Therefore, vestibular-balance symptoms appear to have potentially different pathophysiological features, according to their durations. This potentiality is significant, especially for persistent vestibular-balance symptoms, because they are thought to be related to multiple factors, such as the vestibular system, the somatosensory nervous system, the autonomic nervous system, and psychological factors. Again, we suggest that further studies examine the association between symptom duration and other clinical features. A patient-reported outcome scale that can measure distinctively different durations of vestibular-balance symptoms should be useful in this regard.

Another key finding of the present study is that the two subscales (the VSS-sf-V and VSS-sf-A) were clearly divided with no cross-loading to the other subscale, and that the two factors in the VSS-sf-V (the Vestibular-balance long symptom and the Vestibular-balance short symptom) were not sharply divided. This finding suggests that the two-subscale structure of the VSS-sf may have clear clinical interpretability. Meanwhile, three-factor structures may be useful for the clinical research mentioned above, although two cross-loading items (dizziness lasting all day, difficulty standing or walking) may complicate interpretation. We suggest that further studies examine the dimensional structure in different cultural samples and develop a new patient-reported outcome scale if necessary.

In the present study, we demonstrated that the VSS-sf Japanese version has good psychometric properties overall. We found a slight floor/ceiling effect. The construct validity was considered to be good. The internal consistency and reproducibility were excellent.

The present study has some strengths. First, our sample can be considered to be representative of the target population of individuals who complete the VSS-sf, because the sample cases were recruited based on diagnosis by neuro-otology specialists at multiple sites in various locations. The demographic characteristics and clinical status of the study sample (Table 2) are considered to be similar to those encountered in routine clinical practice at otolaryngology clinics. Second, we performed the cross-cultural adaptation process with sufficient attention to both the semantic and conceptual aspects of the test. Contrary to the recommended guidelines, we chose translators who were neuro-otologists or psychiatrists because the translation of Japanese phrases related to vestibular symptoms requires a degree of familiarity with Japanese patient expressions of vestibular-balance symptoms. To account for the absence of non-medical translators, we modified the Japanese text of the VSS-sf with an emphasis on cognitive interviews with patients and clinical detectivity of therapeutic change. Third, we used the recent recommended methodology for factor analysis, specifically, the method in which the optimal number of factors is determined, thus enhancing the clinical implications of our study.

Several limitations should be taken into account when interpreting the results of the present study. The first limitation concerns the interval between the initial test and the retest. The risk of recall bias cannot be denied because of the short interval. However, our sensitivity analysis of reproducibility showed that this risk was minimal. There are no definite guidelines regarding the optimal interval, which depends on both the target population and the measured concept. We considered the 1 to 3-day interval to be appropriate for the current study population, as in previous studies. Second, we only employed one back-translator although the guideline recommends two independent back-translators [20]. To compensate for this, we employed a native English speaker who advised us regarding the back-translation, ensured sufficient discussion between experts, and conducted cognitive interviews with patients. Third, the present study was conducted for the population in a secondary care context in Japan, using the Japanese version of the VSS-sf. Further research is required in other languages and populations. Finally, we evaluated neither the responsiveness nor interpretability of the VSS-sf in the present study. Further research is required to examine these properties.

## Conclusions

In the present study, we demonstrated that the VSS-sf has a three-factor structure, which includes Vestibular-balance long-duration symptoms, Vestibular-balance short-duration symptoms, and Autonomic-anxiety symptoms. These factors were cross-culturally well-matched with previous studies using the VSS-lv. It was suggested that vestibular-balance symptoms can be analyzed separately according to symptom duration, which might reflect pathophysiological factors. The VSS-sf is useful because it can be used to easily evaluate not only vestibular-balance symptoms and autonomic-anxiety symptoms, but also different durations of vestibular-balance symptoms. Further research using the VSS-sf should be required in other languages and populations.

## Abbreviations

VSS-sf-T/V/A: Vertigo Symptom Scale-short form-Total score/Vestibular-balance subscale/Autonomic-anxiety subscale
VSS-lv: Vertigo Symptom Scale-long version
DHI-T/E/F/P: Dizziness Handicap Inventory-Total score/Emotional subscale/Functional subscale/Physical subscale
HADS-A/D: Hospital Anxiety and Depression Scale-Anxiety subscale/Depression subscale
CTT: Classical Test Theory
IRT: Item Response Theory
CFA: Confirmatory factor analysis
EFA: Exploratory factor analysis
SRMR: Standardized root mean square residual
CFI: Comparative fit index
RMSEA: Root mean square error of approximation
KMO: the Kaiser–Meyer–Olkin measure of sampling adequacy
MAP: Minimum average partial
BIC: Bayesian information criterion
ICC: Interclass correlation coefficients
